# Editorial: Advances in integrated disease management (IDM) for soil-borne plant pathogens: innovative approaches and underlying action mechanism at molecular level

**DOI:** 10.3389/fpls.2023.1256979

**Published:** 2023-08-31

**Authors:** Raja Asad Ali Khan

**Affiliations:** ^1^ Sanya Nanfan Research Institute, Hainan University, Sanya, China; ^2^ Engineering Center of Agricultural Microbial Preparation Research and Development of Hainan, Hainan University, Haikou, China

**Keywords:** pests, pesticides, integrated disease management, diseases, soil-borne

Soil-borne plant pathogens pose a significant threat to plants, and their management is comparatively more difficult than other plant pathogens because of their nature of the hidden enemy, long persistency in soil, vast genetic diversity and host range (Katan, 2017). However, researchers throughout the world are struggling to investigate effective management strategies to manage soil-borne plant pathogens (Panth et al., 2020). We proposed this Research Topic in order to make a useful collection of novel studies which involve the management of soil-borne plant pathogens and underlying action mechanisms. Among soil-borne plant bacterial pathogens, *Ralstonia solanacearum* is one of the most devastating agents affecting different plants in 45 families. Several management practices have been used effectively for the management of this bacterium in different plants, including the use of biological control agents (Wang et al.). However, because of difficulties in handling, culturing, and maintaining biocontrol agents or the issues related to their practical efficacy in soil, the use of their antimicrobial compounds is considered a good alternative. Ye et al. investigated an antibacterial furoic acid compound, 5-(hydroxymethyl)-2-furoic acid, produced by the fungus *Aspergillus niger*. The soil application of this compound effectively controlled the *R. solanacearum* population in soil by direct killing effect as well as enhancing host resistance in tomato plants. Wang et al. reviewed other studies for the management of this bacterium, including biological, organic, breeding, genetic engineering, physical, cultural, chemical and nano-technological approaches.

Nanotechnology emerged as one of the most rapidly advancing sciences of the twenty-first century, and plant scientists revealed the effective application of nanotechnology for the management of plant pathogens. Dutta et al., in their review, focused on several aspects of the use of nanotechnology in the management of soil-borne plant pathogens highlighting the role of nanoparticles as protectants and inducers of plant defense against soil-borne plant pathogens. Uncovering the mechanism of host resistance and discoveries of host genes/proteins that regulate host resistance against soil-borne plant pathogens will help plant genetic engineers develop plants that can better fight against soil-borne plant pathogens. Tian et al. reported that small G-protein StRab5b positively regulates potato resistance against the soil-borne fungus *Phytophthora infestans*. Pazarlar et al. found that *Bacillus cereus* EC9 protects tomatoes against *Fusarium* wilt through JA/ET-activated immunity. In another study by Ling et al., it was shown that *WRKY* genes confer resistance to *Cucumis metuliferus* against root-knot nematodes (RKN). The RKN-resistant variety of *C. metuliferus* (cm3) was found to specifically recruit beneficial bacterial communities in soil upon infestation with RKN, as reported by Song et al. Similarly, Sikandar et al. reviewed overall management strategies, including several recent efforts to improve host resistance against *Meloidogyne enterolobii*.

Enhancing host resistance is also a prominent action mechanism of many biocontrol agents. In their review, Dutta et al. unveil the molecular mechanism by which *Trichoderma* fungus, one of the most used and studied biocontrol fungus, induce host resistance against pathogens including the role of exogenous elicitors in the induction of host resistance. Zhou et al. proved that exogenous application of elicitor isotianil could significantly alleviate the symptoms of *Fusarium* wilt on the banana. They further confirmed that isotianil application might contribute to disease control by inducing host plant defense against *Fusarium* infection. Investigation into how the pathogens achieve virulence at the molecular level is also essential for understanding the action mechanism of the pathogen. Xu et al. revealed the role of cAMP phosphodiesterase in the formation of sclerotia and achieving virulence in *Sclerotinia sclerotiorum*, an important phytopathogenic fungus that causes stem rot and white mold disease.

Most investigations conducted for the management of soil-borne pathogens are based on greenhouse or controlled laboratory conditions. Although the outcomes of these studies provide useful insights into the action mechanism and control efficacy, field investigations are vital for assessing the practical applications of these strategies. Field assessments consider the complex interplay between plants, pathogens and soil, and environmental factors that can affect disease management and development. This more accurately illustrates how these strategies perform in real agricultural scenarios. Moreover, field assessment can give an actual clue of management strategy on economic feasibility.

## Author contributions

RK: Writing – original draft, Writing – review & editing, Conceptualization.
